# S223. COMBINED TREATMENT WITH A SELECTIVE PDE10A INHIBITOR TAK-063 AND ANTIPSYCHOTICS AT SUBEFFECTIVE DOSES PRODUCES POTENT ANTIPSYCHOTIC-LIKE EFFECTS WITHOUT EXACERBATING SIDE EFFECTS PROFILE IN RODENTS

**DOI:** 10.1093/schbul/sby018.1010

**Published:** 2018-04-01

**Authors:** Kazunori Suzuki, Akina Harada, Hirobumi Suzuki, Atsushi Nakatani, Clizia Capuani, Annarosa Ugolini, Mauro Corsi, Nidhi Kaushal, Konstantin Bobkov, John Vekich, Joseph Doyle, Haruhide Kimura

**Affiliations:** 1 Takeda Pharmaceutical Company Limited; 2 Aptuit, Inc

## Abstract

**Background:**

Activation of indirect pathway medium spiny neurons (MSNs) via promotion of cAMP production is the principal mechanism of action (MOA) of current antipsychotics with dopamine D2 receptor antagonism. Phosphodiesterase 10A (PDE10A) inhibitors activate both direct and indirect pathway MSNs by increasing both cAMP and cGMP levels by inhibiting their degradation, which might be expected to promote activation of intracellular signaling similar to that of D2 antagonists in the indirect pathway MSNs. Thus, the activation of the indirect MSN pathway through the distinct MOA of these compounds raises the possibility of augmented pharmacologic effects with combined treatment.

In this study, we compared gene-regulation patterns in the indirect pathway MSNs induced by the PDE10A inhibitors T-773 and T-609, and the D2 antagonist haloperidol, using a cell-type-specific comprehensive gene expression analysis in Drd2-bacTRAP (translating ribosome affinity purification) mice. The pharmacologic effects of combined treatment with another PDE10A inhibitor, TAK-063, and clinically used antipsychotics, haloperidol (HAL) and olanzapine (OLA), were evaluated in multiple rodent models.

**Methods:**

Male ICR mice, Drd2-bacTRAP mice, and Sprague-Dawley rats were used. The indirect pathway MSN-specific gene expression changes by T-773, T-609, and HAL were investigated using RNA sequencing of striatal samples of Drd2-bacTRAP mice. The activation of MSNs in rats was evaluated by measuring glutamate receptor subunit 1 phosphorylation (pGluR1) levels. An in vitro electrophysiological study on the corticostrial pathway in rats was conducted in a slice preparation. The activation of each MSN pathway was assessed by inducing genes as pathway-specific markers: enkephalin for the indirect pathway and substance P for the direct pathway. Suppression of MK-801- or methamphetamine (METH)-induced hyperactivity was assessed by measuring locomotor activity for 2 hours after administration of these stimulants to rats. Improvement of prepulse inhibition (PPI) was investigated in a MK-801-induced PPI deficit mouse model.

**Results:**

Translational profiling in Drd2-bacTRAP mice treated with the PDE10A selective inhibitors, T-773 and T-609, and with HAL suggested regulatory of a largely overlapping signaling pathway by these compounds in the indirect pathway MSNs: 87% of the genes regulated by HAL were also regulated by both T-773 and T-609. Combined treatment with TAK-063 and either HAL or OLA produced an augmented effect on pGluR1 in the rat striatum. An electrophysiological study in rat brain slices indicated that TAK-063 enhanced synaptic responses to a similar extent in both direct and indirect pathway MSNs. Additional evaluation using MSN pathway-specific markers revealed that coadministration of TAK-063 with HAL or OLA additively activated the indirect pathway, but not the direct pathway. Combined treatment with TAK-063 (0.1 mg/kg p.o.) and either HAL (0.3 mg/kg p.o.) or OLA (3 mg/kg p.o.) at subeffective doses produced augmented effects on METH- or MK-801–induced hyperactivity in rats and MK-801–induced PPI deficits in mice. TAK-063 at 0.1 mg/kg did not affect plasma prolactin levels and cataleptic response induced by HAL or OLA in rats.

**Discussion:**

PDE10A inhibitors and HAL showed similar patterns of gene regulation in indirect pathway MSNs in mice. Combined treatment with TAK-063 and either HAL or OLA at subeffective doses produced significant antipsychotic-like effects but no augmentation of the plasma prolactin level and cataleptic response. Although further preclinical and clinical studies will be needed, TAK-063 may provide a novel mechanism as a PDE10A inhibitor for use as combination therapy in schizophrenia.

